# Validando a Inteligência Artificial antes do Uso à Beira do Leito: ChatGPT-4 versus Mini em Conteúdo sobre Hipertensão Baseado em Diretrizes

**DOI:** 10.36660/abc.20260254

**Published:** 2026-05-05

**Authors:** Cláudio Tinoco Mesquita, Fernanda Azevedo Silva, Ana Beatriz Costa do Couto

**Affiliations:** 1 Universidade Federal Fluminense Niterói RJ Brasil Universidade Federal Fluminense, Niterói, RJ – Brasil; 2 Hospital Universitário Antônio Pedro Niterói RJ Brasil Hospital Universitário Antônio Pedro, EBSERH, Health, Science & Education Lab, Niterói, RJ – Brasil; 3 Universidade Federal Fluminense Programa de Pós-Graduação em Ciências Cardiovasculares Niterói RJ Brasil Programa de Pós-Graduação em Ciências Cardiovasculares – Universidade Federal Fluminense, Niterói, RJ – Brasil; 4 Hospital Pró-Cardíaco Rio de Janeiro RJ Brasil Hospital Pró-Cardíaco, Rio de Janeiro, RJ – Brasil

**Keywords:** Inteligência Artificial, ChatGPT, Hipertensão, Diretrizes Clínicas, Validação

A integração da inteligência artificial (IA) na prática médica é uma realidade. Desde o suporte à tomada de decisão clínica e o desenho de estudos até ferramentas de chatbots e a produção de materiais educativos, os modelos de linguagem de grande escala (LLMs) são cada vez mais utilizados na prática clínica. Nesse contexto, estudos como o de Ataídes et al.,^1^ que avaliam a performance de materiais educacionais baseados em LLMs, são fundamentais.

A hipertensão é uma das principais causas de morte prematura; sendo este um dos motivos que fazem a Organização Mundial da Saúde (OMS) estimular o desenvolvimento de campanhas de prevenção. Trata-se de uma doença que afeta múltiplos órgãos, causando grande impacto em todo o organismo. A condição é definida por pressão arterial sistólica ≥140 mmHg ou pressão arterial diastólica ≥90 mmHg, de acordo com as diretrizes da Sociedade Brasileira de Cardiologia.^2^ O número de indivíduos diagnosticados com hipertensão aumenta a cada ano, e estudos indicam que há grande influência dos hábitos alimentares da população, que atualmente são caracterizados por alto consumo de alimentos ultraprocessados.^3^

O estudo que comparou ChatGPT-mini e ChatGPT-4.0 na geração de conteúdo educacional sobre hipertensão aborda um aspecto muito importante da IA na medicina: a qualidade e a segurança das informações direcionadas aos pacientes. Ao avaliar sistematicamente acurácia, completude, qualidade estrutural (EQIP), consistência e alinhamento com diretrizes clínicas, os autores realizaram uma avaliação abrangente dos conteúdos educacionais que foram gerados por IA.^1^

À primeira vista, comparar versões de modelos de LLMs pode parecer algo sensível ao tempo, em especial em um campo caracterizado por rápida evolução tecnológica. Na verdade, em fevereiro de 2026, os modelos de LLMs baseados no GPT-4 foram definitivamente descontinuados no ecossistema do ChatGPT, sendo substituídos por gerações mais recentes (GPT-5). No entanto, isso não diminui a relevância do estudo. Pelo contrário, reforça sua mensagem central.

**Figura 1:**
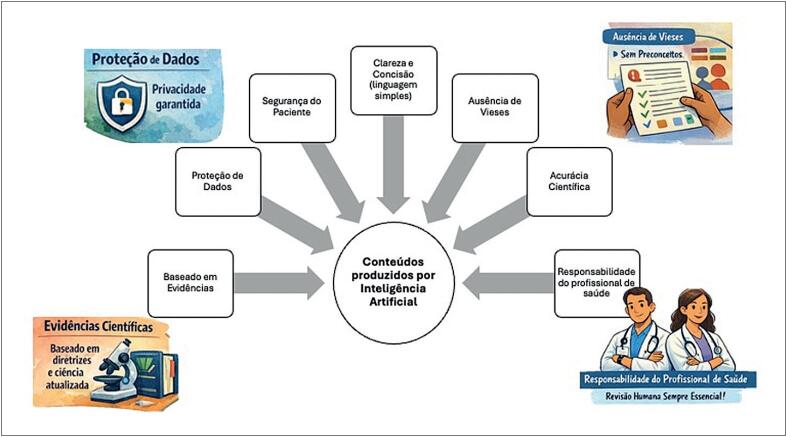
Esta figura resume os principais domínios para avaliar conteúdos gerados por IA para pacientes: acurácia científica e base em evidências, revisão por especialista e responsabilidade, clareza e legibilidade, ausência de vieses, segurança do paciente e privacidade de dados. Em conjunto, esses elementos reforçam que os conteúdos produzidos por IA exigem validação sistemática e supervisão humana antes do uso clínico.

Os autores devem ser parabenizados, pois o verdadeiro valor do trabalho não está em determinar qual modelo apresenta melhor desempenho, mas em demonstrar que nenhum conteúdo gerado por IA deve ser considerado confiável sem validação rigorosa.^4^ Mesmo modelos avançados, capazes de produzir textos fluentes, coerentes e aparentemente confiáveis, podem gerar imprecisões, omitir informações críticas ou apresentar recomendações desalinhadas com diretrizes estabelecidas.^5^ De forma importante, este estudo reforça um princípio fundamental que deve ser seguido independentemente do modelo ou versão de LLM: a responsabilidade pelo conteúdo oferecido aos pacientes é atribuição dos profissionais de saúde e as instituições — e não responsabilidade do algoritmo^4,6^ (Figura 1).

Por fim, nunca é excessivo reforçar, o uso ético da IA é essencial, especialmente quando LLMs são utilizados no cuidado ao paciente. Os pacientes devem ser informados quando o conteúdo for produzido com auxílio da IA, e as instituições devem estabelecer processos claros de revisão, aprovação e atualização periódica dos materiais educativos.

Em conclusão, embora as tecnologias continuem a evoluir, o princípio fundamental permanece inalterado: antes que a IA alcance o paciente, ela deve ser cuidadosamente testada, validada e contextualizada.
