# Supplementary material for: Validando a Inteligência Artificial antes do Uso à Beira do Leito: ChatGPT-4 versus Mini em Conteúdo sobre Hipertensão Baseado em Diretrizes
Source: Arq Bras Cardiol. 2026 May 5;123(2):e20260254. [Article in Portuguese] doi: 10.36660/abc.20260254 (PMC13155790; doi:10.36660/abc.20260254)
Supplement: Translation

# Translation: Validating Artificial Intelligence before the Bedside: ChatGPT-4 vs Mini in Guideline-Based Hypertension Content

**DOI:** 10.36660/abc.20260254i

**Authors:** Cláudio Tinoco Mesquita, Fernanda Azevedo Silva, Ana Beatriz Costa do Couto

**Affiliations:** 1 Niterói RJ Brazil Universidade Federal Fluminense, Niterói, RJ – Brazil; 2 Niterói RJ Brazil Hospital Universitário Antônio Pedro, EBSERH, Health, Science & Education Lab, Niterói, RJ – Brazil; 3 Niterói RJ Brazil Programa de Pós-Graduação em Ciências Cardiovasculares – Universidade Federal Fluminense, Niterói, RJ – Brazil; 4 Rio de Janeiro RJ Brazil Hospital Pró-Cardíaco, Rio de Janeiro, RJ – Brazil

**Keywords:** Artificial Intelligence, ChatGPT, Hypertension, Clinical Guidelines, Validation

The rapid integration of artificial intelligence (AI) into medical practice is a reality. From clinical decision support and study design to chatbot tools and the production of educational materials, large language models (LLMs) are increasingly used in daily practice. In this context, studies like the study of Ataídes et al.,^1^ evaluating the performance of LLM-based materials, are crucial.

Hypertension is one of the leading causes of premature death; therefore, the World Health Organization (WHO) invests in prevention campaigns. It is a disease that affects multiple organs, causing a major impact on the entire body. The condition is defined by a systolic blood pressure (BP) of 140 mmHg or higher, or a diastolic BP of 90 mmHg, according to Brazilian Society of Cardiology guidelines.^2^ The number of individuals diagnosed with hypertension increases each year, and studies indicate that the population's eating habits are characterized by high consumption of ultra-processed foods.^3^

The study comparing ChatGPT-mini and ChatGPT-4.0 in the generation of hypertension educational content addresses a very important aspect of AI in medicine: the quality and safety of patient-directed information. By systematically assessing accuracy, completeness, structural quality (EQIP), consistency, and alignment with clinical guidelines, the authors provided a comprehensive framework for evaluating AI-generated health content.^1^

At first glance, comparing two specific model versions may appear time-sensitive in a field characterized by rapid technological turnover. Indeed, as of February 2026, GPT-4-based models have been fully discontinued within the ChatGPT ecosystem, with newer generations now in use. However, this does not diminish the relevance of the study. On the contrary, it reinforces its central message.

The authors must be congratulated because the true value of their work lies not in determining which model performs better, but in demonstrating that no AI-generated content should be assumed reliable without rigorous validation.^4^ Even advanced models capable of producing fluent, coherent, and seemingly authoritative text may generate inaccuracies, omit critical information, or present recommendations that are misaligned with established guidelines.^5^ Importantly, this study underscores a very important principle that must be followed with any specific LLM model or version: the responsibility for the content delivered to patients remains with healthcare professionals and institutions—not with the algorithm^4,6^ (Figure 1).

**Figure 1:** This figure summarizes the main domains to assess AI-generated content for patients: scientific accuracy and evidence base, expert review and responsibility, clarity and readability, absence of bias, patient safety, and data privacy. Together, these elements reinforce that AI outputs require systematic validation and human oversight before clinical use.

Finally, ethical use of AI is critical, especially when LLMs are used in patient management. Patients should be informed when content is AI-assisted, and institutions must establish clear processes for review, approval, and periodic updating of educational materials^7^.

In conclusion, while technologies will continue to evolve, the fundamental requirement remains unchanged: before AI reaches the patient, it must be carefully tested, validated, and contextualized.
